# *MC1R* Is a Prognostic Marker and Its Expression Is Correlated with MSI in Colorectal Cancer

**DOI:** 10.3390/cimb43030108

**Published:** 2021-10-11

**Authors:** Lixiong Peng, Jiang Chang, Xilin Liu, Shiying Lu, Honglin Ren, Xiaoshi Zhou, Zengshan Liu, Pan Hu

**Affiliations:** 1 Key Laboratory of Zoonosis Research, Ministry of Education, Institute of Zoonosis, College of Veterinary Medicine, Double-First Class Discipline of Human-Animal Medicine, Jilin University, Changchun 130015, China; penglx1996@163.com (L.P.); jiangchang2017@163.com (J.C.); lushiying1129@163.com (S.L.); renhl@jlu.edu.cn (H.R.); zhouxs1996@163.com (X.Z.); zsliu1959@sohu.com (Z.L.); 2 Norman Bethune Health Science Center, Jilin University, Changchun 130015, China; liuxilin@jlu.edu.cn

**Keywords:** *MC1R*, CRC, SNPs, clinicopathologic features, prognostic marker

## Abstract

Melanocortin 1 receptor (*MC1R*) is thought to be a marker of poor prognosis and a potential target for the treatment of melanoma. Studies have found that *MC1R* promotes several tumor behaviors, including cell proliferation and differentiation, pigment formation, and genome damage repair. Some single-nucleotide polymorphisms (SNPs) of *MC1R* are involved in the occurrence and development of melanoma. A few studies have reported a relationship between *MC1R* and colorectal cancer (CRC). In this research, our objective was to examine *MC1R* expression and *MC1R* SNPs and investigate their correlation with the clinicopathological features of human CRC tissues. We evaluated *MC1R* mRNA expression by performing bioinformatic analyses on human CRC expression datasets. We used Western blotting and RT-qPCR to compare *MC1R* expression in CRC tissues with that in normal tissues, and *MC1R* SNPs in CRC tissues were detected by PCR-direct sequencing (DS). The expression of *MC1R* was significantly decreased in CRC tissues compared with normal tissue, and its expression was negatively associated with *P53* expression, *MLH1* expression, and *PMS2* expression, and high *MC1R* expression was significantly associated with microsatellite instability (MSI). *MC1R* SNPs were also associated with the clinicopathological characteristics of CRC; for example, the rs2228479 locus genotype was correlated with *Ki67* status, and the rs885479 locus genotype was correlated with age and T stage. In conclusion, *MC1R* plays a crucial role in the progression of CRC and may be a marker of poor prognosis in CRC.

## 1. Introduction

Colorectal cancer (CRC) is a frequently diagnosed cancer worldwide, with high mortality rates. It is estimated that 1.15 million new cases occurred and 0.58 million patients died of CRC worldwide in 2020 [1]. Currently, *B-Raf proto-oncogene* *(BRAF), CEA cell adhesion molecule 5(CEA), epidermal growth factor receptor (EGFR)*, and *KRAS proto-oncogene (KRAS)* are the most reliable prognostic biomarkers of CRC [2,3,4]. *Phosphatidylinositol 3-kinase (PI3K)*, *tumor protein p53 (TP53)*, and *methyltransferase 14 (METTL14)* are also potential prognostic markers of CRC and can be used as markers of tumor occurrence, metastasis, survival, or recurrence [5,6,7]. Although many molecules can provide guidance for the prognosis and treatment of CRC, most advanced CRC patients are currently difficult to cure, and seeking a novel biomarker for synergistic or independent diagnosis and prognosis provides a basis for new drug screening and personalized treatment.

*Melanocortin 1 receptor (*MC1R*)* is a cell surface endocytic receptor with seven transmembrane domains that belong to the G-protein-coupled receptor family; *MC1R* is composed of 317 amino acids and is present in both the cell membrane and the cytoplasm [8]. *Human *MC1R** may perform physiological functions with its ligand *melanocyte stimulator (α-MSH)* [9]. Under normal circumstances, *α-MSH-*MC1R** participates in the regulation of diverse signaling pathways, including cell proliferation and differentiation, pigment formation, antioxidation, and repair of genome damage [10]. Abnormal expression of *MC1R* is associated with the development of skin cancer, and it has been reported that high expression of *MC1R* in melanoma promotes the progression of its development. Thus, *MC1R* is thought to be associated with a poor prognosis and a potential target for the treatment of melanoma, and many studies have shown that the treatment of melanoma by targeting *MC1R* achieves good efficacy [11,12].

A broad range of genetic studies have shown that *MC1R* is a highly polymorphic gene, and some of its are single nucleotide polymorphisms (SNPs) [8,13]. *MC1R* variants are recognized genetic risk factors for many diseases, and some SNPs are closely related to human hair color, skin color, and skin cancer [14]. For example, *MC1R* variants have been found in most red-haired people in Europe, and studies show that these people are more likely to suffer from skin cancer [15]. Currently, approximately 100 *MC1R* SNPs have been found in melanoma, although the significance of most SNPs is not clear, and approximately 10 of these SNPs have been shown to be important for the occurrence and development of melanoma and can be used for its diagnosis [16,17,18].

Mismatch repair (MMR) is an important DNA repair mechanism, which can accurately identify and repair the base mismatch produced during DNA replication or recombination [19]. Microsatellite instability (MSI), referring to the phenomenon of altered MS sequence length due to insertion or deletion mutations during DNA replication, is often caused by defects in MMR function [20]. Therefore, detecting the loss of MMR genes including *MLH1*, *MSH2*, *MSH6*, and *PMS2* is one of the methods for determining whether MSI has occurred in CRC [21]. Studies have shown that the MSI assay can be used to effectively assess the benefits of anti-*PD1* immunotherapy in CRC therapy [21].

To our knowledge, few studies have reported the relationship between *MC1R* and the occurrence and development of CRC. In our research, we studied the expression of *MC1R* and *MC1R* SNPs and their correlation with clinicopathological features in human CRC tissues to analyze whether *MC1R* is a diagnostic or prognostic biomarker of CRC. Our results showed that the expression of *MC1R* was significantly lower in CRC tissue than in normal tissue, and the expression of *MC1R* was significantly associated with the status of the MSI. Furthermore, two SNPs of *MC1R*, rs2228479 and rs885479, were also associated with the clinicopathological features of CRC. Thus, we can conclude that *MC1R* is associated with the occurrence and development of CRC and is a potential prognostic biomarker for CRC.

## 2. Materials and Methods

### 2.1. Bioinformatic Analysis

#### 2.1.1. RNA-Sequencing Data and Samples

Raw counts of *MC1R* RNA-sequencing data and corresponding clinical information from 620 CRC patients were obtained from The Cancer Genome Atlas (TCGA) dataset (https://portal.gdc.cancer.gov/, accessed on 18 April 2021). Ten colorectal normal tissue samples were obtained from TCGA, and 779 normal tissue samples were obtained from the GTEx V8 release version (https://gtexportal.org/home/datasets, accessed on 18 April 2021). Data from GSE147571, comprising 308 CRC patients, and GSE44076, comprising 98 CRC tumor samples and 98 paired normal samples, were obtained from the GEO database (https://www.ncbi.nlm.nih.gov/geo/, accessed on 6 May 2021).

#### 2.1.2. Significant Prognostic Marker Analysis and Correlation Analysis of *MC1R* and Clinicopathological Features

We used R software version v4.0.3 (The R Foundation for Statistical Computing, 2020) with the limma, ggrisk, forestplot, rms, and ggplot2 packages for the significant prognostic marker analysis and correlation analysis of *MC1R* and clinicopathological features.

#### 2.1.3. Correlation Analysis of *MC1R* and Mismatch Repair (MMR) Genes or Differential Genes

Two-gene correlation graphs were generated with the R software package ggstatsplot. The differential expression of mRNAs was studied by using the limma package of R software. The adjusted P value was analyzed to correct for false-positive results in the GEO datasets. “Adjusted *p* < 0.05 and log (fold change) >1 or log (fold change) < −1” were defined as the thresholds for the screening of differential expression of mRNAs. Multigene correlation graphs were displayed by the R software package pheatmap.

#### 2.1.4. Construction of *MC1R* and the Related Genes Network

We performed protein–protein interaction (PPI) network analysis between *MC1R* and associated differential genes and four MMR genes by building a regulatory network using Cytoscape software, and we analyzed it using STRING (https://string-db.org/, accessed on 27 May 2021).

### 2.2. Patients and Clinical Tissue Samples

A total of 86 colorectal cancer tumor samples and 83 normal samples were collected after surgical treatment in the China-Japan Union Hospital (Changchun, China) from 2015 to 2021, including 61 pairs of paired tumor and normal tissue samples. The paired normal tissue we obtained came from noncancerous regions at least 5 cm from the edge of the tumor. The fourth edition of the World Health Organization (WHO) Classification of tumors of the digestive system (Fiori 2013) was used for the histological classification. The seventh edition of the AJCC *Cancer Staging Manual* (Benedix et al., 2013; Edge and Compton 2010) was used to assess the differentiation grade and TNM stage of CRC. This research was approved by the Protection of Ethics Committee of China-Japan Union Hospital (2019012803). Informed consent was obtained from all the participants included in the study.

### 2.3. Protein Extraction and Western Blotting Assay

Proteins of different tissue samples were extracted using RIPA lysis buffer (Beyotime, Shanghai, China) containing 1% PMSF (Solarbio, Beijing, China), and their levels were measured with the BCA protein assay kit (Solarbio, Beijing, China).

Thirty micrograms of protein were denatured in sample buffer and then electrophoresed on 10% SDS–PAGE. The PVDF membranes (Immobilin, Carrigtwohill, Ireland) were incubated with primary antibodies (1:400 dilution of *MC1R* antibody (Immunoway, Tennyson Pkwy Ste 250, Plano, USA); 1:1000 dilution of *β-actin* antibody (Cell Signaling Technology, Denvers, Massachusetts, USA) overnight at 4 °C after electrophoresis, transfer, and blockage. Next, a secondary antibody (1:4000 dilution of goat anti-rabbit (Immunoway, Tennyson Pkwy Ste 250, Plano, USA)) was added at room temperature for 1 h. Then, the immunoblots were marked using ECL (Solarbio, Beijing, China). Finally, based on densitometry and ImageJ Software, analyses were performed in triplicate, and *MC1R* expression was presented as the grayscale value of *MC1R* compared to the corresponding *β-actin* in tissue.

### 2.4. RNA Extraction and Reverse Transcription

A 50 mg piece of CRC tumor tissue or normal tissue was cut and collected in an RNase-free tube with 250 μL DEPC water and 3 steel balls. After that, TissueLyser-II (Retsch, Germany) was used to break the tissue blocks, and the supernatant was collected in a new RNase-free tube. Finally, 900 μL of TRIzol reagent (Invitrogen, 5791 Van Allen Way, CA, USA) was added to an RNase-free tube, and the supernatant was stored. The following steps were used to extract total mRNA according to the manufacturer’s instructions.

Total mRNA was reverse-transcribed to cDNA using a PrimeScript™ RT reagent Kit with gDNA Eraser (Takara, Kusatsu, Japan). The first step to remove the genomic DNA involved 2 μL of 5× gDNA Eraser Buffer, 1 μL of gDNA Eraser, 1 μg of total mRNA, and DEPC water for a total volume of 10 μL, which was placed at 42 °C for 2 min to complete the response. The second step, the reverse transcription response, involved 10 μL of reaction solution in the first step, 1 μL of PrimeScript RT Enzyme Mix I, 1 μL of RT Primer Mix, and RNase-Free dH2O for a total volume of 20 μL, which was placed at 42 °C for 15 min, then 85 °C for 6 s to complete the response. The RT product was placed at −80 °C for short-term storage.

### 2.5. Quantitative Real-Time PCR Analysis (RT-qPCR)

FastStart Universal SYBR Green Master Mix (ROX) on an ABI 7500 Real-Time PCR System was used to amplify cDNA through quantitative real-time PCR. The amplification reactions contained 2 µL of diluted RT product, 0.5 µL of objective mRNA forward primer, 0.5 µL of objective mRNA reverse primer, 10 µL of 2× M5 HiPer Real-time PCR Super mix with Low Rox (Mei5 Biotechnology, MF797, Beijing, China), and 7 µL RNase-free water. The PCR cycling conditions were 95 °C for 5 min followed by 40 cycles at 95 °C for 20 s and 60 °C for 50 s. *MC1R* expression was normalized to human *β-actin* expression, and the experiment was performed in triplicate. By means of the ∆∆Ct equation, the expression of *MC1R* in clinical tissue samples was calculated in comparison with the endogenous control *β-actin*. Related primers are shown below: *MC1R*, 5′-GCTACCACAGCATCGTGACC-3′ (forward primer) and 5′-ACGTGGTCGTAGTAGGCGAT-3′ (reverse primer); *β-actin*, 5′-CATGTACGTTGCTATCCAGGC-3′ (forward primer) and 5′-CTCCTTAATGTCACGCACGAT-3′ (reverse primer).

### 2.6. Genomic DNA Extraction and SNPs Analysis

A TIANamp Genomic DNA Kit (Tiangen, DP304-02, Beijing, China) was used to extract genomic DNA from CRC tumor samples according to the manufacturer’s instructions. Using an epoch multivolume spectrophotometer system (BioTek, VT, Retsch, Germany), DNA purity and quantity were determined.

The coding region of *MC1R* was amplified using I-5™ 2× High-Fidelity Master Mix (MCLAB, I5HM-100, Oyster Point Boulevard, San Francisco, USA) and a PCR amplifier (SCILOGEX, TC1000-G, Texas, USA). The primers used to amplify the *MC1R* coding region were as follows: 5′-CCTCCAACG ACTCCTTCCTGCTTC-3′ (forward primer) and 5′-ACAATATCACCACCTCCCT CTGC -3′ (reverse primer). The PCR product was recovered by a TIANgel Midi Purification Kit (Tiangen, DP209-02, Beijing, China) and sequences were analyzed by ABI 3730XL DNA analyzer (Massachusetts, USA). BioEdit Analysis software was used to analyze the resulting data, and the results were validated by comparison with the NCBI gene bank.

### 2.7. Statistical Analysis

**Figure 1:**
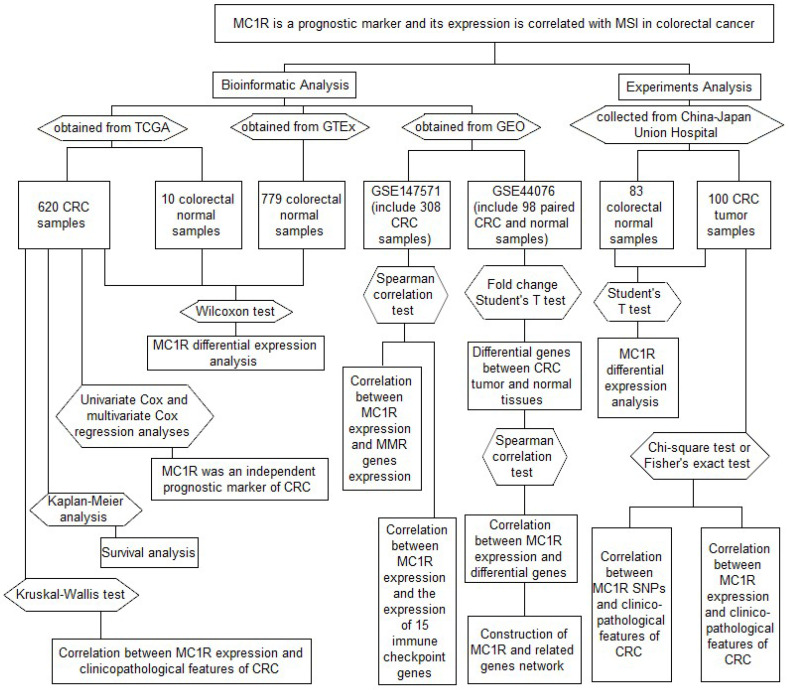
Illustration of study workflow. The flowchart of data collection and method implementation in this work.

R scripts/Bioconductor packages, SPSS 25.0, and GraphPad Prism 8.0 were used for statistical data analyses and figures. We performed univariate Cox analysis and multivariate Cox regression analysis to investigate whether *MC1R* can effectively predict the prognosis of CRC. The Wilcoxon test and Kruskal–Wallis test were used to compare *MC1R* expression in different groups. After dividing CRC patients into the *MC1R* high-expression group and *MC1R* low-expression group, we constructed survival curves of CRC by Kaplan–Meier analysis. Student’s *t* test was used to analyze the data for *MC1R* expression, which we detected using the Western blotting assay and RT-qPCR assay. The correlations between *MC1R* and the clinicopathological features and the correlations between *MC1R* SNPs and the clinicopathological features were examined by chi-square test or Fisher’s exact test. The correlation between *MC1R* expression and targets of interest, including MMR genes, the tumor microenvironment, immune checkpoint genes, immune cells, and differential genes, was evaluated by the Spearman correlation test. All statistical results with *p* < 0.05 were considered to be statistically significant. The data collection and method implementation in this study were summarized in flowchart (Figure 1).

## 3. Results

### 3.1. *MC1R* Is an Independent Marker for CRC Prognosis, and High *MC1R* Expression Is Associated with Advanced T, N, and TNM Stage

**Figure 2:**
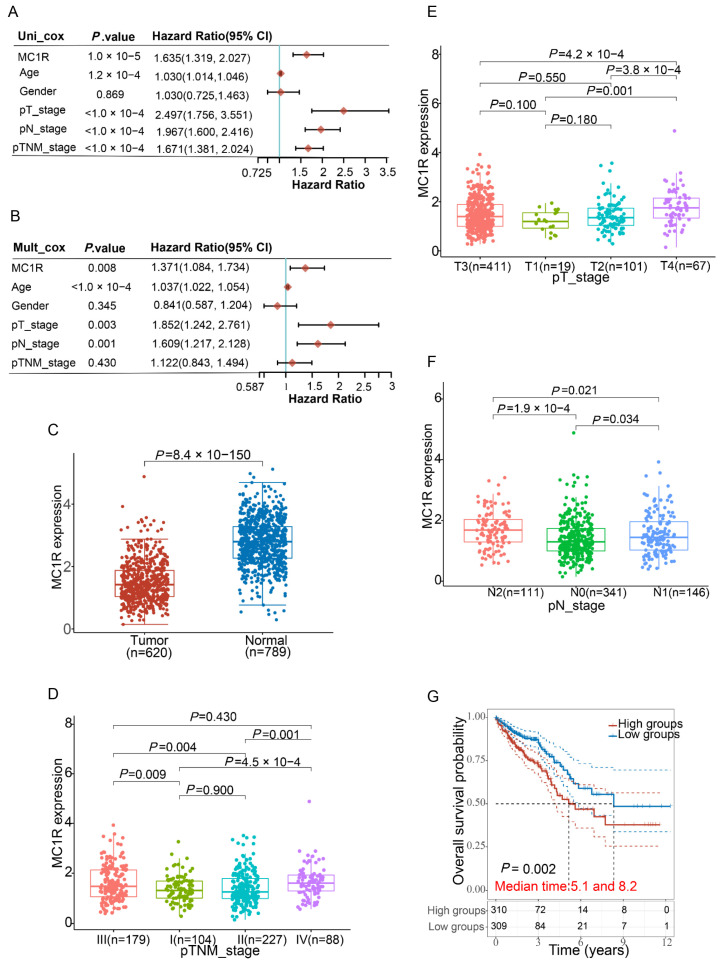
Prediction of the prognostic significance of *MC1R* in CRC and differential expression of *MC1R* in CRC. (**A**) and (**B**) Hazard ratio and P value of constituents involved in univariate and multivariate Cox regression and some features of the *MC1R* genes. (**C**) The expression of *MC1R* in colorectal cancer is significantly lower than that in normal tissue. Correlation between *MC1R* expression and clinicopathological features of CRC, including (**D**) T stage, (**E**) N stage, and (**F**) TNM stage. (**G**) Kaplan–Meier curves show overall survival among patients with CRC stratified by *MC1R* expression.

To explore whether *MC1R* was an independent prognostic marker of CRC, the forest approach was used to show the P value, HR, and 95% CI of each variable through the forestplot R package. We found that age, T stage, N stage, TNM stage, and *MC1R* expression level were significantly associated with CRC prognosis (*p* < 0.01) by univariate Cox regression analysis; however, sex was not (Figure 2A). Multivariate Cox regression analysis showed that age, T stage, N stage, and *MC1R* were independent prognostic factors (*p* < 0.05); however, sex and TNM stage were not (Figure 2B).

RNA-sequencing data comprising 620 CRC patients obtained from TCGA, 10 colorectal normal tissue samples obtained from TCGA, and 779 normal tissue samples obtained from the GTEx V8 release version were used to study the differential expression of *MC1R* mRNA levels between CRC tumor samples and normal samples. The results showed a significant decrease (*p* < 0.0001) in the mRNA level of *MC1R* in CRC tumor samples in comparison to normal samples (Figure 2C).

To explore the correlation between *MC1R* and CRC, data from 598 CRC tumor samples collected from TCGA were employed to analyze the correlation between *MC1R* expression and the clinicopathological features of CRC by the Wilcox test and Kruskal test. Analysis showed that *MC1R* expression significantly correlated with the T stage (*p* < 0.01, Figure 2D), N stage (*p* < 0.05, Figure 2E), and TNM stage (*p* < 0.01, Figure 2F) of CRC. These results showed that *MC1R* was more highly expressed in advanced T, N, and TNM stages. Furthermore, the survival curve also showed that high *MC1R* expression was associated with a shorter 5-year survival time and higher mortality in CRC (Figure 2G).

Taken together, these findings demonstrated that *MC1R* is an independent marker of a CRC prognosis.

### 3.2. *MC1R* Expression Is Significantly Decreased in CRC Tumor Tissue

**Figure 3:**
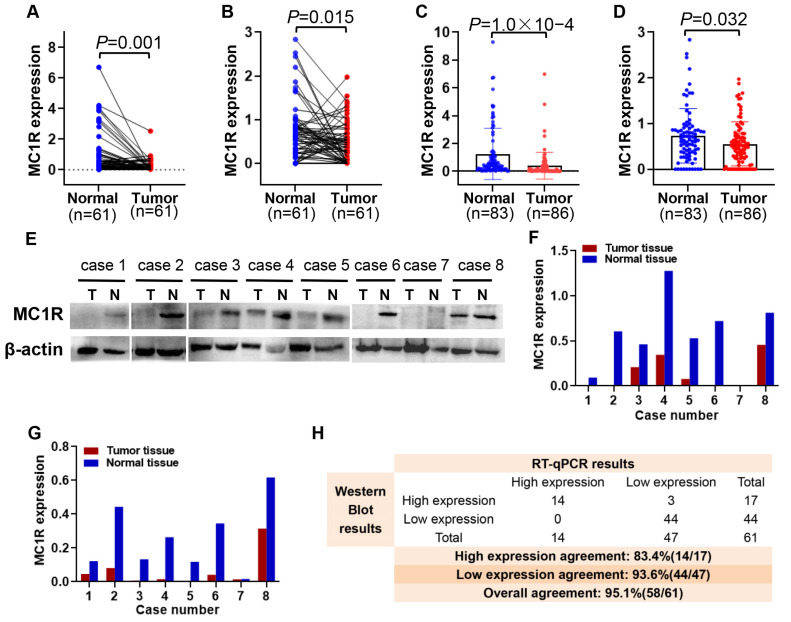
*MC1R* expression in CRC clinical tumor samples. *MC1R* expression in 61 CRC tumor samples and paired normal tissue samples (**A**) Detected by RT-qPCR (**B**) Detected by Western blotting. *MC1R* expression in 92 CRC tumor samples and 85 normal tissue samples. (**C**) Detected by RT-qPCR (**D**) Detected by Western blotting. *MC1R* expression in 8 representative cases that belong to the results of 61 paired CRC and adjacent normal tissues. (**E**) and (**F**) Detected by Western blotting (**G**) Detected by RT-qPCR. (**H**) Consistency analysis for detecting *MC1R* by RT-qPCR and Western blotting in tissue samples.

In this part, we examine the expression of *MC1R* at the protein and mRNA levels in 86 CRC tumor tissues and 83 adjacent normal tissues by RT-PCR and Western blotting, respectively. The data for *MC1R* expression were analyzed with Student’s *t* test, and the results showed that both the expression of *MC1R* at the protein and mRNA levels were significantly lower in CRC tumor samples (*N* = 61) than in paired normal tissues (*p* < 0.05) (Figure 3A,B). The trend of downregulation was also observed when comparing unpaired tumor samples and all normal samples (*p* < 0.05) (Figure 3C,D). Here, we present the detection results of eight more representative cases (Figure 3E–G). Forty-seven (77.0%) and forty-four (72.1%) cases showed that *MC1R* was expressed at lower levels in CRC tumor samples than in normal tissue by RT-qPCR and Western blotting, respectively. In addition, the overall agreement was 95.1% (58/61) (Figure 3H). Although the two approaches had different truncation points, their tendency of *MC1R* expression was the same, and both indicated that *MC1R* was expressed at lower levels in CRC tumor samples than in normal samples.

### 3.3. *MC1R* Expression Is Associated with P53 Expression and MSI

*MC1R* expression in a total of 86 CRC tumor samples was detected to verify the correlation between *MC1R* expression and the clinicopathological features of CRC obtained from bioinformatic analysis, as well as to more extensively investigate the prognostic value of *MC1R* in association with clinicopathological features. The median value of *MC1R* expression was used as the division basis. The patients were divided into an *MC1R* high expression group and an *MC1R* low expression group and analyzed using the corresponding pathological information. The results are shown in Table 1.

**Figure 4:**
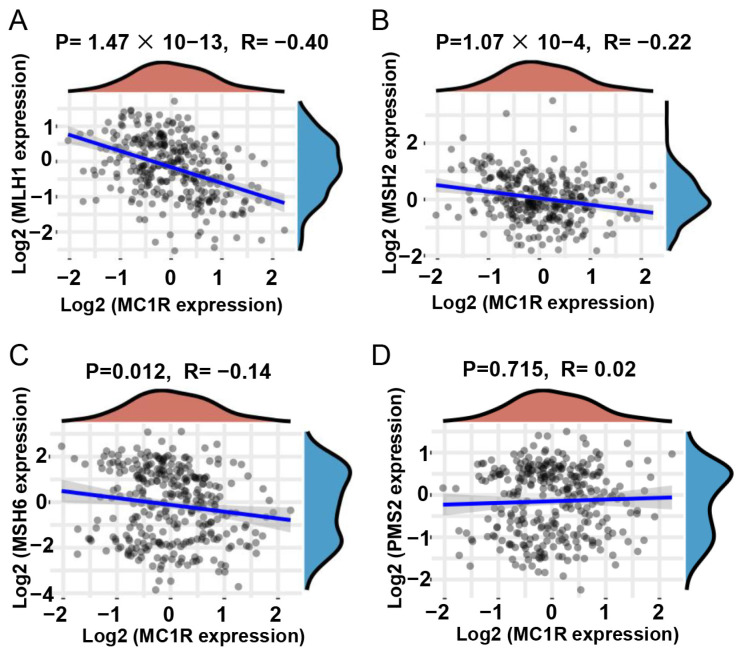
Correlation between *MC1R* expression and MMR genes expression. (**A**) *MLH1*, (**B**) *MSH2*, (**C**) *MSH6*, and (**D**) *PMS2* relative to *MC1R* expression.

Univariate analysis using the chi-square test revealed that *MC1R* expression was significantly correlated with *P53* status (*p* = 0.030), *MLH1* status (*p* = 0.048), *PMS2* status (*p* = 0.041), and MS status (*p* = 0.034) in the clinical CRC samples that we examined. To further verify that the expression of *MC1R* was correlated with MS status, GSE147571 was used to analyze the correlation between *MC1R* expression and MMR genes, including *MLH1, MSH2, MSH6*, and *PMS2*. The results of the bioinformatics analysis show that *MC1R* expression was significantly and negatively correlated with *MLH1* (*p* = 1.47 × 10^−13^, R = −0.40) (Figure 4A), *MSH2* (*p* = 1.07 × 10^−4^, R = −0.22) (Figure 4B), and *MSH6* (*p* = 0.012, R = −0.14) (Figure 4C) expression but not significantly correlated with *PMS2* expression (*p* = 0.715, R = 0.02) (Figure 4D). Surprisingly, this result was substantially consistent with the analysis of clinical CRC tumor samples, and both analyses indicated that high *MC1R* expression was more likely correlated with MMR genes, which further suggested that high *MC1R* expression was significantly associated with MSI.

### 3.4. *MC1R* Is Associated with Immune Cell Infiltration and Immune Checkpoint Genes

**Figure 5:**
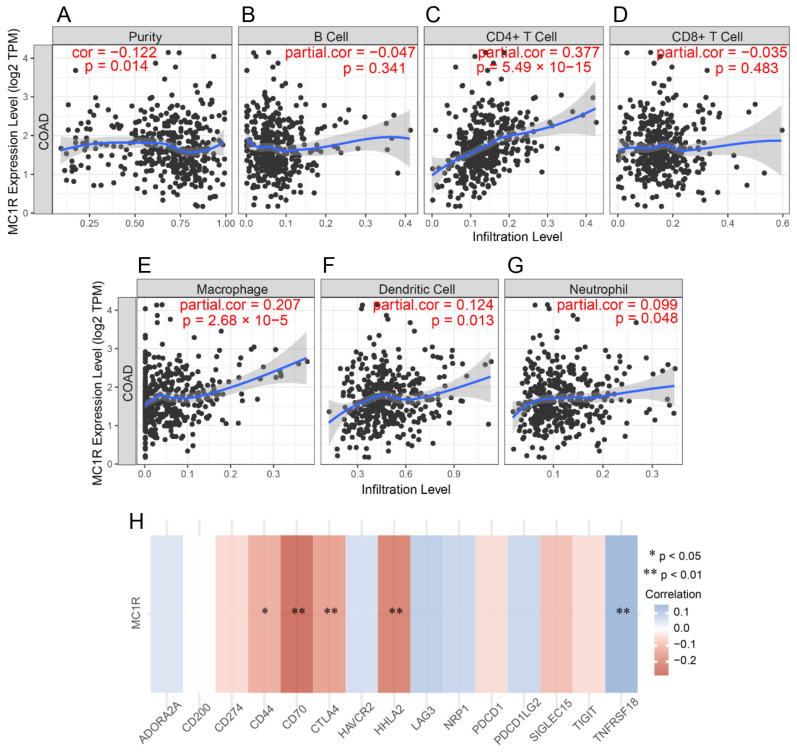
Immunological features related to *MC1R* in CRC. Correlation analysis of *MC1R* expression and infiltration of immune cells in CRC in the TCGA cohort. (**A**) Tumor purity, (**B**) B cells, (**C**) CD4+ T cells, (**D**) CD8+ T cells, (**E**) macrophages, (**F**) neutrophils, and (**G**) dendritic cells relative to *MC1R* expression. (**H**) Correlation between *MC1R* expression and the expression of 15 immune checkpoint genes.

CRC is a highly heterogeneous type of cancer, and its progression and immunotherapy can be influenced by the tumor microenvironment and immune cell infiltration. We wanted to explore whether *MC1R* expression was associated with immune cell infiltration. The results showed a positive correlation with CD4 + T cells (r = 0.377, *p* = 5.49 × 10^−15^, Figure 4C), macrophage cells (r = 0.207, *p* = 2.68 × 10^−5^, Figure 5E), dendritic cells (r = 0.124, *p* = 1.27 × 10^−2^, Figure 5F), and neutrophil cells (r = 0.099, *p* = 4.84 × 10^−2^, Figure 5G) and a negative significant correlation between *MC1R* and tumor homogeneity (r = −0.122, *p* = 1.42 × 10^−2^, Figure 5A).

To investigate whether *MC1R* is associated with immune checkpoint genes in CRC, GSE147571 CRC data were used to assess the correlation between *MC1R* and 15 immune checkpoint genes. We found that *MC1R* was significantly correlated with *CD44* (*p* < 0.05, r = −0.13), *CD70* (*p* < 0.01, r = −0.28), *CTLA4* (*p* < 0.01, r = −0.16), *HHLA2* (*p* < 0.01, r = −0.23), and *TNFRSF18* (*p* < 0.01, r = 0.15) (Figure 5H).

### 3.5. Sequencing Results of *MC1R* Polymorphisms

**Figure 6:**
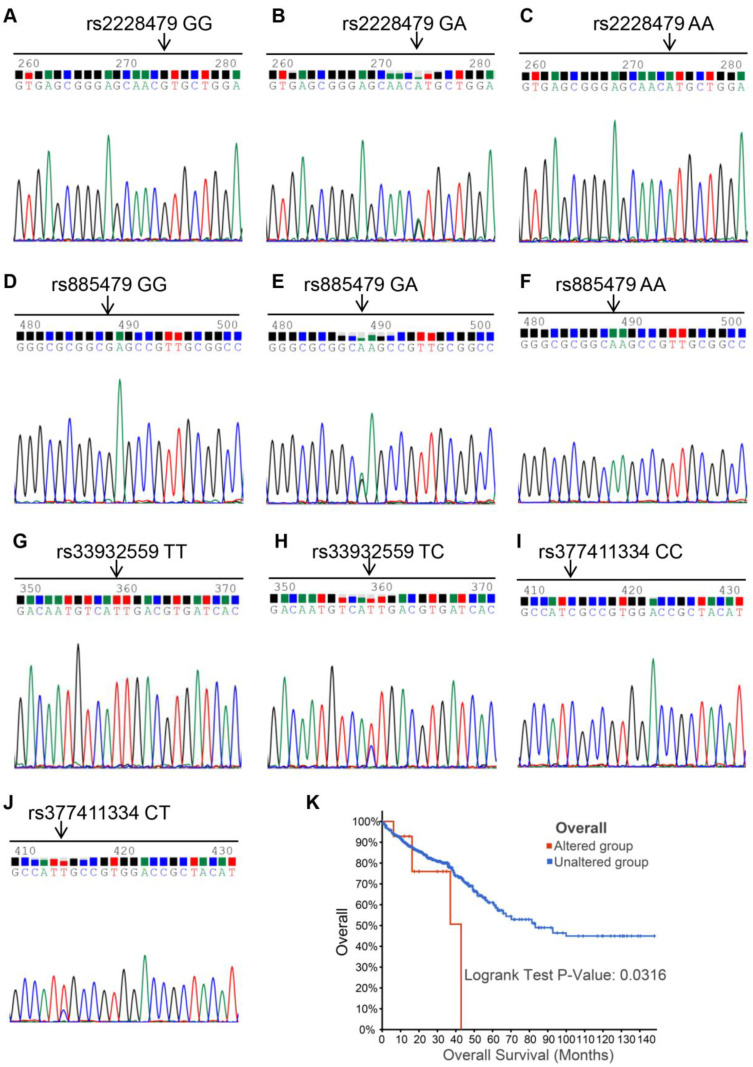
Chromatograph of sequencing of *MC1R* coding sequence. (**A**) homozygotic type of SNP locus rs2228479; (**B**) and (**C**) heterozygous type of SNP locus rs2228479; (**D**) homozygotic type of SNP locus rs885479; (**E**) and (**F**) heterozygous type of SNP locus rs885479; (**G**) homozygotic type of SNP locus rs33932559; (**H**) heterozygous type of SNP locus rs33932559; (**I**) homozygotic type of SNP locus rs377411334; (**J**) heterozygous type of SNP locus rs377411334. (**K**) Overall survival of 15 *MC1R* altered group and 1476 *MC1R* unaltered group patients in the Kaplan–Meier Plotter.

*MC1R* has many gene polymorphisms, and 100 CRC patient samples were used to detect whether specific SNP mutations exist in CRC. The sequencing results identified four SNP mutations: rs2228479, rs885479, rs33932559, and rs377411334. The genotypes of the four SNP loci were GG/GA/AA, GG/GA/AA, TT/TC, and CC/CT (Figure 6A–J). Allele frequencies were 83.0% (GG, 83/100), 13.0% (GA, 13/100), and 4.0% (AA, 4/100) for the SNP locus rs2228479, 10.0% (GG, 10/100), 36.0% (GA, 36/100), and 54.0% (AA, 54/100) for the SNP locus rs885479, 99.0% (TT, 99/100) and 1.0% (TC, 1/100) for the SNP locus rs33932559, and 99.0% (CC, 99/100) and 1.0% (CT, 1/100) for the SNP locus rs377411334.

### 3.6. Correlation Analysis of *MC1R* SNP Polymorphisms and Clinicopathological Features

There were no data concerning whether *MC1R* polymorphisms could be used as a tumor prognosis factor to predict the survival of CRC patients. rs33932559 and rs377411334 were synonymous mutations, but rs2228479 and rs885479 were nonsynonymous mutations. In addition, allele frequencies for rs2228479 (17.0%) and rs885479 (90%) were more than 15%. Thus, we selected the two SNP loci mentioned above to confirm the role of *MC1R* gene polymorphisms in the prognosis of patients. We analyzed the relations between *MC1R* SNP polymorphisms and clinical parameters, and the results are shown in Table 2.

The overall survival of 15 *MC1R* altered group patients and 1476 *MC1R* unaltered group patients in the Kaplan–Meier Plotter Database from cBioPortal (www.cbioportal.org, accessed on 5 June 2021) showed that the *MC1R* altered group was associated with an unfavorable prognosis in CRC patients (Figure 6K). The rs2228479 locus genotype was not correlated with sex, age, differentiation, T stage, N stage, TNM stage, *P53* status, *MLH1* status, *MSH2* status, *MSH6* status, *PMS2* status, or MS status (*p* > 0.05; Table 2) and was only correlated with *Ki67* status (*p* > 0.01; Table 2). The rs885479 locus genotype was correlated with age (*p* > 0.05; Table 2) and T stage (*p* > 0.01; Table 2) but not with sex, differentiation, N stage, TNM stage, *P53* status, *Ki67* stage, *MLH1* status, *MSH2* status, *MSH6* status, *PMS2* status, or MS status (*p* > 0.05; Table 2).

### 3.7. *MC1R* Is Associated with the Regulation of Cell Population Proliferation

**Figure 7:**
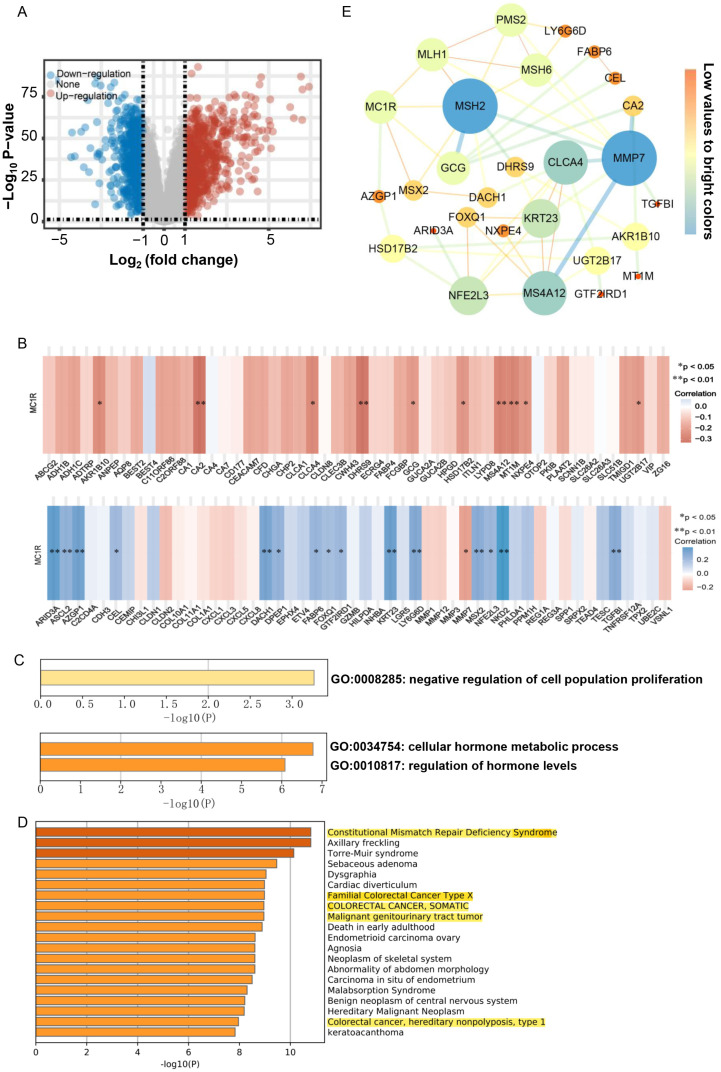
Gene Set Enrichment Analysis of *MC1R* in CRC. (**A**) Volcano plots were constructed using fold-change values and adjusted P. (**B**) Heat map of the correlation between *MC1R* and differential genes. The correlation between *MC1R* and the 50 upregulated genes with the largest difference change (above). The correlation between *MC1R* and the 50 downregulated genes with the largest difference change (below). (**C**) GO enrichment analysis for positively associated differential genes (above); GO enrichment analysis for adversely associated differential genes (below). (**D**) Summary of enrichment analysis in DisGeNET12 for *MC1R* and associated differential genes and four MMR genes. (**E**) The network for *MC1R*, four MMR genes, and the 26 correlated differential genes. In this figure, the size and gradient color of the node are adjusted by degree, and the edge thickness and gradient color are adjusted by the combined score.

GSE44076 was used for bioinformatic analysis to further investigate the genes potentially associated with *MC1R* in CRC. First, we performed differential gene expression analysis based on the Limma package (version: 3.40.2) of R software between 98 CRC tumor samples and paired normal adjacent mucosa samples. Our analysis identified a total of 1579 genes as significantly regulated genes, which contained 816 upregulated genes and 718 downregulated genes (Figure 7A). Second, we chose the 50 most significantly upregulated genes and the 50 most significantly downregulated genes, and their expression in CRC tumor tissues and paired normal tissues is shown in Supplementary (Figure S1). Next, we analyzed the correlation between *MC1R* expression and the expression of the above 100 differentially expressed genes by Spearman correlation analysis. The results show that *MC1R* expression was significantly associated with 26 differential genes. The genes *ARID3A* (r = 0.376, *p* = 0.000), *ASCL2* (r = 0.260, *p* = 0.010), *AZGP1* (r = 0.345, *p* = 0.001), *CEL* (r = 0.219, *p* = 0.030), *DACH1* (r = 0.330, *p* = 0.001), *DPEP1* (r = 0.253, *p* = 0.012), *FABP6* (r = 0.240, *p* = 0.017), *FOXQ1* (r = 0.203, *p* = 0.045), *GTF2IRD1* (r = 0.226, *p* = 0.025), *KRT23* (r = 0.357, *p* = 0.000), *LY6G6D* (r = 0.301, *p* = 0.003), *MSX2* (r = 0.282, *p* = 0.005), *NFE2L3* (r = 0.234, *p* = 0.021), *NKD2* (r = 0.393, *p* = 6.29 × 10^−5^), and *TGFBI* (r = 0.282, *p* = 0.005) were positively associated with the expression of *MC1R* (Figure 7B), and genes including *MMP7* (r = −0.231, *p* = 0.022), *AKR1B10* (r = −0.248, *p* = 0.014), *CA2* (r = −0.326, *p* = 0.001), *CLCA4* (r = −0.255, *p* = 0.011), *DHRS9* (r = −0.326, *p* = 0.002), *GCG* (r = −0.207, *p* = 0.041), *HSD17B2* (r = −0.218, *p* = 0.031), *MS4A12* (r = −0.272, *p* = 0.007), *MT1M* (r = −0.267, *p* = 0.008), *NXPE4* (r = −0.222, *p* = 0.028), and *UGT2B17* (r = −0.209, *p* = 0.039) were adversely associated with the expression of *MC1R* (Figure 7B).

Then, gene ontology (GO) enrichment analysis using the associated differentially expressed genes showed that the genes positively associated with *MC1R* expression were enriched in the negative regulation of cell proliferation and that the genes adversely associated with *MC1R* expression were enriched in the regulation of hormone levels and cellular hormone metabolic processes (Figure 7C). In addition, *MC1R* and associated differentially expressed genes and four MMR genes were used for enrichment analysis in DisGeNET12. The results were enriched in Constitutional Mismatch Repair Deficiency Syndrome, Familial Colorectal Cancer Type X, COLORECTAL CANCER, SOMATIC, Malignant genitourinary tract tumor, colorectal cancer, and hereditary nonpolyposis type 1 (Figure 7D), which further indicated that *MC1R* was associated with the occurrence and development of CRC. Through the constructed PPI network, the interaction between *MC1R* and the above-associated genes was more clearly demonstrated, where *MC1R* may interact directly with *MLH1, MSH2, MSX2, GCG*, and *AZGP1* and thus participate in the development of CRC (Figure 7E). Overall, these findings suggest that *MC1R* might have a role in the progression of CRC.

## 4. Discussion

The correlation between *MC1R* and melanoma has been studied extensively, but the value of *MC1R* in the prognosis or therapeutic potential of CRC has been investigated to a lesser extent [22,23]. Studies have recently shown that *MC1R* participates in the occurrence and development of CRC [24]. However, the expression of *MC1R* in CRC and its role in the tumorigenesis of CRC remain unclear. In our study, we explored the correlation between *MC1R* and CRC.

In our results, bioinformatic analysis indicated that *MC1R* was expressed at lower levels in CRC than in normal tissue. We explored the expression of *MC1R* in clinical samples and found that *MC1R* expression was lower in CRC tumor tissues than in normal tissues at both the mRNA and protein levels. However, we found that high *MC1R* expression was associated with malignancy or later stages of CRC using bioinformatic analysis. The survival curve also indicated that high *MC1R* expression was associated with higher mortality in CRC. There are many differences between cancer and normal tissues, the involvement of *MC1R* with other molecules in the regulation of organism function in cancer and normal tissues may also be inconsistent, and the specific reasons remain to be further explored. These controversial results confuse the role of *MC1R* in the initiation or progression of CRC. Some studies have shown that *MC1R* expression or its splicing variants could predict unfavorable prognosis in melanoma patients [25,26]. We did not find a significant correlation between *MC1R* expression and the above clinicopathological features of CRC in the clinical samples we analyzed. The reasons for these discrepancies may be multifactorial. Histologic subtypes may account for some of the discrepancies. However, we found that *MC1R* expression was significantly and negatively correlated with *P53* expression, *MLH1* expression, and *PMS2* expression and that high *MC1R* expression was significantly associated with MSI. The *P53* gene is one of the most commonly inactivated tumor inhibitors in human cancer. The function of *P53* in the progression of cancer is associated with a variety of transcription and nontranscriptional activities that lead to strict control of cell proliferation and death, senescence, and DNA repair [27]. In our research, *MC1R* expression was significantly associated with *P53* expression, suggesting that *MC1R* may be directly or indirectly associated with *P53* and then jointly involved in the process of colorectal cancer. Thus, given these findings, we can infer that *MC1R* may be an unfavorable prognostic marker for CRC.

Studies have recently shown that *MC1R* is an immune-related gene in CRC [28,29]. MSI and tumor microenvironments are effective biomarkers for a variety of tumor immunotherapy responses. Association analyses between *MC1R* and MSI revealed that *MC1R* influences this biomarker. MSI has been detected in approximately 15% of all CRCs and is a hypermutable phenotype caused by the loss of DNA MMR activity that can be used to guide clinical immunotherapy for the treatment of CRC [30]. One of the determination methods of MSI is to detect the expression of *MLH1, MSH2, MSH6,* and *PMS2* proteins in CRC [31]. A study has shown that the *MC1R* gene is a DNA-damage- and DNA-repair-related gene in CRC [24]. Thus, combined with our experimental results and the studies above, we speculate that *MC1R* may participate in the development of CRC by influencing cAMP-mediated DNA damage and the DNA repair response [32]. Furthermore, we found that *MC1R* was significantly associated with immune cell infiltration, such as CD4+ T cells, macrophages, dendritic cells, and neutrophil cells. Immune cells are the basis of immunotherapy; therefore, understanding immune infiltration in the tumor microenvironment is a key to improving response rates and developing novel immunotherapy strategies in tumor therapy [33]. The immune checkpoint refers to a number of inhibitory signaling pathways present in the immune system, and use of some inhibitory signaling pathways to inhibit T cell activity is an important mechanism for tumors to escape immune attack [34]. In recent years, immune checkpoint inhibitors have achieved a great effect in the treatment of cancer, so targeting immune checkpoints has broad applications in anti-cancer therapy [35]. Our results also show that *MC1R* was correlated with immune checkpoint genes, including *CD44, CD70, CTLA4, HHLA2,* and *TNFRSF18*. These findings suggest that the differential expression of *MC1R* may be associate with immunotherapy in CRC.

*MC1R* is a highly polymorphic gene—most polymorphisms are caused by SNPs—and is associated with skin phenotypes and increased cancer risk [36]. Studies show that some SNPs of *MC1R* are involved in the occurrence and development of melanoma, so we explored whether *MC1R* SNPs are associated with CRC. In our research, we did not find new or specific SNP mutations of *MC1R* in CRC but identified four SNP mutations, rs2228479, rs885479, rs33932559, and rs377411334. Rs2228479 contributes to facial pigmented spots and increases the risk of developing late-onset Alzheimer’s disease. Rs885479 was associated with the diagnosis of depression and melanoma rates, and both rs2228479 and rs885479 are correlated with red hair [37,38,39]. Although they have not been reported in CRC, the above study showed that *MC1R* SNPs were closely correlated with the disease, which prompted us to explore whether *MC1R* SNPs were associated with CRC. rs33932559 and rs377411334 were less frequently reported in CRC, and the significance of those associations was unclear, although their mutation rate (1%) was low in our clinical samples; thus, we only analyzed the correlation between the two other *MC1R* SNPs and clinicopathological characteristics of CRC patients. Our results showed that the rs2228479 locus genotype was correlated with *Ki67* status and that the rs885479 locus genotype was correlated with age and T stage. *Ki67* is a marker of cell proliferation and is closely correlated with the degree of differentiation, infiltration, metastasis, and prognosis of many tumors [40]. Coincidentally, when we used differentially expressed genes that were positively associated with *MC1R* for GO enrichment, the enrichment pathway involved negative regulation of cell proliferation. Studies have shown that *MC1R* is involved in regulating cell proliferation through the receptor-γ pathway and that diminished cell proliferation capacity is associated with the upregulation of *MC1R* [41,42]. Therefore, we speculate that one of the ways that *MC1R* participates in the development of colorectal cancer is by regulating the proliferation of tumor cells.

To further investigate the molecular mechanisms underlying the role of *MC1R* in CRC, we searched for associations between *MC1R* and differential genes and constructed a PPI network. The results showed that *MC1R* may interact with *MLH1, MSH2, MSX2, GCG*, and *AZGP1*. *MSX2* was found to be highly expressed in CRC tumor tissues, cell proliferation and invasion were suppressed, cell cycle arrest and apoptosis were promoted, and Akt phosphorylation was inactivated when *MSX2* expression was knocked down [43]. *AZGP1* is a useful diagnostic biomarker found in the tissues and serum of Chinese CRC patients and it promotes epithelial-mesenchymal transition (EMT) in colorectal cancer via the filamin A-mediated focal adhesion pathway [44]. Furthermore, other genes associated with *MC1R* in the PPI, such as *MMP7, CLCA4, MS4A12, KRT23*, and *NFE2L3*, have been shown to play a vital role in the progression of CRC [45,46,47,48,49]. Overall, *MC1R* may be involved in the occurrence and development of CRC by interacting with the above molecules.

To our knowledge, our study is the first to analyze the expression of *MC1R*, SNP locus of the *MC1R* gene in CRC, and their correlation with clinicopathological features in human CRC tissues. However, the small sample size is an intrinsic limitation of this study that did not provide sufficient power to study the relationship we mentioned above. Large-scale clinical studies are needed to evaluate the prognostic correlation of *MC1R* and its SNPs. In addition, detailed understanding of the role of *MC1R* in CRC requires further in vivo and in vitro experiments.

In conclusion, our comprehensive analysis revealed four important results. First, the expression of *MC1R* was significantly decreased in CRC. Second, *MC1R* participated in the development of colorectal cancer by regulating the proliferation of tumor cells, and its expression was associated with *P53* expression. Third, *MC1R* expression was associated with *MLH1* expression, *PMS2* expression, and the status of MS. Fourth, *MC1R* SNPs were also associated with the development of colorectal cancer; for example, the rs2228479 locus genotype was correlated with *Ki67* status, and the rs885479 locus genotype was correlated with age and T stage. Thus, *MC1R* plays an important role in the progression of CRC and may be a potential prognostic marker for CRC.

## Figures and Tables

**Table 1:** Relationship between *MC1R* expression and clinicopathological characteristics of CRC patients.

Clinicopathological Characteristics	Total Patients	*MC1R*		*p*
		*MC1R* Low Expression Group	*MC1R* High Expression Group	
Total Sex	86	43	43	
				0.639
Male	60	31 (51.7%)	29 (48.3%)	
Female	26	12 (46.2%)	14 (53.8%)	
Age				0.826
≥65	35	18 (51.4%)	17 (48.6%)	
<65	51	25 (49.0%)	26 (51.0%)	
Differentiation				0.655
Middle	54	28 (51.9%)	26 (48.1%)	
Poor	32	15 (46.9%)	17 (53.1%)	
T stage				0.360
T1and T2	5	1 (20.0%)	4 (80.0%)	
T3and T4	81	42 (51.9%)	39 (48.1%)	
N stage				0.514
N0	49	26 (53.1%)	23 (46.9%)	
N1, N2 and N3	37	17 (45.9%)	20 (54.1%)	
M stage				1.000
M0	1	0 (0.0%)	1 (100.0%)	
M1	85	43 (50.6%)	42 (49.4%)	
TNM stage				0.829
I and II	47	24 (51.1%)	23 (48.9%)	
III and IV	39	19 (48.7%)	20 (51.3%)	
*P53* status				0.030 *
≥70.0%	38	24 (63.2%)	14 (36.8%)	
<70.0%	48	19 (39.6%)	29 (60.4%)	
*Ki67* status				0.079
≥70.0%	65	36 (55.4%)	29 (44.6%)	
<70.0%	21	7 (33.3%)	14 (66.7%)	
*MLH1* status				0.048 *
≥70.0%	64	36 (56.3%)	28 (43.7%)	
<70.0%	22	7 (31.8%)	15 (68.2%)	
*MSH2* status				0.213
≥70.0%	74	39 (52.7%)	35 (47.3%)	
<70.0%	12	4 (33.3%)	8 (66.7%)	
*MSH6* status				0.268
≥70.0%	70	37 (52.9%)	33 (47.1%)	
<70.0%	16	6 (37.5%)	10 (62.5%)	
*PMS2* status				0.041 *
≥70.0%	66	37 (56.1%)	29 (43.9%)	
<70.0%	20	6 (30.0%)	14 (70.0%)	
MS status				0.034 *
MSI	18	5 (27.8%)	13 (72.2%)	
MSS	68	38 (55.9%)	30 (44.1%)	

Analysis software: SPSS 25.0 statistical software; analysis method: Chi-square test or Fisher’s exact test. * *p* < 0.05 was considered significant.

**Table 2:** Correlation analysis of *MC1R* SNPs and clinical factors.

Clinical Factors	Total Patients	rs2228479		*p*	rs885479		*p*
		GG (%)	GA and AA (%)		GG (%)	GA and AA (%)	
Total	100						
Sex Male	70	57 (81.4)		0.772			0.274
			13 (18.6)		9 (12.9)	61 (87.1)	
Female	30	26 (86.7)	4 (13.3)		1 (3.3)	29 (96.7)	
Age				0.514			0.013 *
≥65	40	32 (80.0)	8 (20.0)		8 (20.0)	32 (80.0)	
<65	60	51 (85.0)	9 (15.0)		2 (3.3)	58 (96.7)	
Differentiation				0.913			0.738
Middle	60	50 (83.3)	10 (16.7)		6 (10.0)	54 (90.0)	
Poor	40	33 (82.5)	7 (17.5)		3 (7.5)	37 (92.5)	
T stage				1.000			0.001 *
T1and T2	6	5 (83.3)	1 (16.7)		4 (66.7)	2 (33.3)	
T3 and T4	94	78 (83.0)	16 (17.0)		6 (6.4)	88 (93.6)	
N stage				0.661			1.000
N0	54	44 (81.5)	10 (18.5)		5 (9.3)	49 (91.7)	
N1, N2 and N3	46	39 (84.8)	7 (15.2)		5 (10.9)	41 (90.1)	
TNM stage				0.661			0.506
I and II	54	44 (81.5)	10 (18.5)		4 (7.4)	50 (92.6)	
III and IV	46	39 (84.8)	7 (15.2)		6 (13.0)	40 (87.0)	
Total	86						
*P53* status				0.913			1.000
≥70.0%	38	32 (84.2)	6 (15.8)		3 (7.9)	35 (92.1)	
<70.0%	48	40 (83.3)	8 (16.7)		4 (8.3)	44 (91.7)	
*Ki67* status				0.004*			1.000
≥70.0%	65	59 (90.8)	6 (9.2)		5 (7.7)	60 (92.3)	
<70.0%	21	13 (61.9)	8 (38.1)		2 (9.5)	19 (90.5)	
*MLH1* status				0.336			1.000
≥70.0%	64	55 (85.9)	9 (14.1)		5 (7.8)	59 (92.2)	
<70.0%	22	17 (77.3)	5 (22.7)		2 (9.1)	20 (90.9)	
*MSH2* status				1.000			0.251
≥70.0%	74	62 (83.8)	12 (16.2)		5 (6.8)	69 (93.2)	
<70.0%	12	10 (83.3)	2 (16.7)		2 (16.7)	10 (83.3)	
*MSH6* status				1.000			0.610
≥70.0%	70	58 (82.9)	12 (17.1)		5 (7.1)	65 (92.9)	
<70.0%	16	14 (87.5)	2 (12.5)		2 (12.5)	14 (87.5)	
*PMS2* status				0.299			1.000
≥70.0%	66	57 (86.4)	9 (13.6)		6 (9.1)	60 (90.9)	
<70.0%	20	15 (75.0)	5 (25.0)		1 (5.0)	19 (95.0)	
MS status				1.000			0.633
MSI	18	15 (83.3)	3 (16.7)		2 (11.1)	16 (88.9)	
MSS	68	57 (83.8)	11 (16.2)		5 (7.4)	63 (92.6)	

Analysis software: SPSS 25.0 statistical software; analysis method: Chi-square test or Fisher’s exact test. * *p* < 0.05 was considered significant.

## Data Availability

Data is contained within the article or Supplementary Materials.

## Supplementary Materials

The following are available online at https://www.mdpi.com/article/10.3390/cimb43030108/s1.

## References

[1] Ferlay J., Colombet M., Soerjomataram I., Parkin D.M., Piñeros M., Znaor A., Bray F. (2021). Cancer statistics for the year 2020: An overview. Int. J. Cancer.

[2] Corcoran R.B., André T., Atreya C.E., Schellens J.H., Yoshino T., Bendell J.C., Hollebecque A., McRee A.J., Siena S., Middleton G. (2018). Combined *BRAF, EGFR,* and *MEK* Inhibition in Patients with *BRAF*(V600E)-Mutant Colorectal Cancer. Cancer Discov..

[3] Gonzalez-Exposito R., Semiannikova M., Griffiths B., Khan K., Barber L.J., Woolston A., Spain G., Von Loga K., Challoner B., Patel R. (2019). *CEA* expression heterogeneity and plasticity confer resistance to the *CEA*-targeting bispecific immunotherapy antibody cibisatamab (*CEA*-TCB) in patient-derived colorectal cancer organoids. J. Immunother. Cancer.

[4] Lal N., White B.S., Goussous G., Pickles O., Mason M., Beggs A., Taniere P., Willcox B.E., Guinney J., Middleton G.W. (2017). *KRAS* Mutation and Consensus Molecular Subtypes 2 and 3 Are Independently Associated with Reduced Immune Infiltration and Reactivity in Colorectal Cancer. Clin. Cancer Res..

[5] Papadatos-Pastos D., Rabbie R., Ross P., Sarker D. (2015). The role of the *PI3K* pathway in colorectal cancer. Crit. Rev. Oncol..

[6] Terradas M., Mur P., Belhadj S., Woodward E.R., Burghel G.J., Munoz-Torres P.M., Quintana I., Navarro M., Brunet J., Lazaro C. (2020). *TP53*, a gene for colorectal cancer predisposition in the absence of Li-Fraumeni-associated phenotypes. Gut.

[7] Yang X., Zhang S., He C., Xue P., Zhang L., He Z., Zang L., Feng B., Sun J., Zheng M. (2020). *METTL14* suppresses proliferation and metastasis of colorectal cancer by down-regulating oncogenic long non-coding RNA XIST. Mol. Cancer.

[8] Horrell E.M.W., Boulanger M.C., D’Orazio J.A. (2016). *Melanocortin 1 Receptor*: Structure, Function, and Regulation. Front. Genet..

[9] Chen S., Zhu B., Yin C., Liu W., Han C., Chen B., Liu T., Li X., Chen X., Li C. (2017). Palmitoylation-dependent activation of *MC1R* prevents melanomagenesis. Nature.

[10] Herraiz C., Garcia-Borron J.C., Jiménez-Cervantes C., Olivares C. (2017). *MC1R* signaling. Intracellular partners and pathophysiological implications. Biochim. et Biophys. Acta (BBA)-Mol. Basis Dis..

[11] Tafreshi N.K., Tichacek C.J., Pandya D.N., Doligalski M.L., Budzevich M.M., Kil H., Bhatt N.B., Kock N.D., Messina J.L., Ruiz E.E. (2019). *Melanocortin 1 Receptor*-Targeted alpha-Particle Therapy for Metastatic Uveal Melanoma. J. Nucl. Med..

[12] Li M., Liu D., Lee D., Kapoor S., Gibson-Corley K., Quinn T.P., Sagastume E.A., Mott S.L., Walsh S.A., Acevedo M.R. (2019). Enhancing the Efficacy of *Melanocortin 1 Receptor*-Targeted Radiotherapy by Pharmacologically Upregulating the Receptor in Metastatic Melanoma. Mol. Pharm..

[13] Latreille J., Ezzedine K., Elfakir A., Ambroisine L., Gardinier S., Galan P., Hercberg S., Gruber F., Rees J., Tschachler E. (2009). *M**C1R* gene polymorphism affects skin color and phenotypic features related to sun sensitivity in a population of French adult women. Photochem. Photobiol..

[14] Sulem P., Gudbjartsson D., Stacey S.N., Helgason A., Rafnar T., Magnusson K.P., Manolescu A., Karason A., Palsson A., Thorleifsson G. (2007). Genetic determinants of hair, eye and skin pigmentation in Europeans. Nat. Genet..

[15] Hoiom V., Tuominen R., Kaller M., Lindén D., Ahmadian A., Månsson-Brahme E., Egyhazi S., Sjöberg K., Lundeberg J., Hansson J. (2009). *MC1R* variation and melanoma risk in the Swedish population in relation to clinical and pathological parameters. Pigment Cell Melanoma Res..

[16] Guida S., Bartolomeo N., Zanna P.T., Grieco C., Maida I., De Summa S., Tommasi S., Guida M., Azzariti A., Foti C. (2015). Sporadic melanoma in South-Eastern Italy: The impact of *melanocortin 1 receptor (*MC1R*)* polymorphism analysis in low-risk people and report of three novel variants. Arch. Dermatol. Res..

[17] Johansson P.A., Pritchard A.L., Patch A.M., Wilmott J.S., Pearson J.V., Waddell N., Scolyer R.A., Mann G.J., Hayward N.K. (2017). Mutation load in melanoma is affected by *MC1R* genotype. Pigment Cell Melanoma Res..

[18] Tagliabue E., Gandini S., Bellocco R., Maisonneuve P., Newton-Bishop J., Polsky D., Lazovich D., Kanetsky P.A., Ghiorzo P., Gruis N.A. (2018). *MC1R* variants as melanoma risk factors independent of at-risk phenotypic char-acteristics: A pooled analysis from the M-SKIP project. Cancer Manag. Res..

[19] Gupta D., Heinen C.D. (2019). The mismatch repair-dependent DNA damage response: Mechanisms and implications. DNA Repair (Amst).

[20] Baretti M., Le D.T. (2018). DNA mismatch repair in cancer. Pharmacol. Ther..

[21] Dudley J.C., Lin M.T., Le D.T., Eshleman J.R. (2016). Microsatellite Instability as a Biomarker for *PD-1* Blockade. Clin. Cancer Res..

[22] Budden T., Bowden N.A. (2019). *MC1R* CpG island regulates *MC1R* expression and is methylated in a subset of melanoma tumours. Pigment Cell Melanoma Res..

[23] Wendt J., Mueller C., Rauscher S., Fae I., Fischer G., Okamoto I. (2018). Contributions by *MC1R* Variants to Melanoma Risk in Males and Females. JAMA Dermatol..

[24] Wang X.-Q., Xu S.-W., Wang W., Piao S.-Z., Mao X.-L., Zhou X.-B., Wang Y., Wu W.-D., Ye L.-P., Li S.-W. (2021). Identification and Validation of a Novel DNA Damage and DNA Repair Related Genes Based Signature for Colon Cancer Prognosis. Front. Genet..

[25] Chen F., Zhang X., Ma K., Madajewski B., Benezra M., Zhang L., Phillips E., Turker M.Z., Gallazzi F., Penate-Medina O. (2018). *Melanocortin-1 Receptor*-Targeting Ultrasmall Silica Nanoparticles for Dual-Modality Human Melanoma Imaging. ACS Appl. Mater. Interfaces.

[26] Fargnoli M.C., Gandini S., Peris K., Maisonneuve P., Raimondi S. (2010). *MC1R* variants increase melanoma risk in families with CDKN2A mutations: A me-ta-analysis. Eur. J. Cancer.

[27] Lacroix M., Riscal R., Arena G., Linares L.K., Le Cam L. (2019). Metabolic functions of the tumor suppressor *p53*: Implications in normal physiology, metabolic disorders, and cancer. Mol. Metab..

[28] Chen H., Luo J., Guo J. (2020). Development and validation of a five-immune gene prognostic risk model in colon cancer. BMC Cancer.

[29] Ma X.-B., Xu Y.-Y., Zhu M.-X., Wang L. (2021). Prognostic Signatures Based on Thirteen Immune-Related Genes in Colorectal Cancer. Front. Oncol..

[30] Lin A., Zhang J., Luo P. (2020). Crosstalk Between the MSI Status and Tumor Microenvironment in Colorectal Cancer. Front. Immunol..

[31] Vilar E., Gruber S.B. (2010). Microsatellite instability in colorectal cancer-the stable evidence. Nat. Rev. Clin. Oncol..

[32] Jarrett S.G., Carter K.M., Bautista R.M., He D., Wang C., D’Orazio J.A. (2018). *Sirtuin 1*-mediated deacetylation of XPA DNA repair protein enhances its interaction with ATR protein and promotes cAMP-induced DNA repair of UV damage. J. Biol. Chem..

[33] Zhang Y., Zhang Z. (2020). The history and advances in cancer immunotherapy: Understanding the characteristics of tumor-infiltrating immune cells and their therapeutic implications. Cell. Mol. Immunol..

[34] Waldman A.D., Fritz J.M., Lenardo M.J. (2020). A guide to cancer immunotherapy: From T cell basic science to clinical practice. Nat. Rev. Immunol..

[35] Darvin P., Toor S.M., Sasidharan N.V., Elkord E. (2018). Immune checkpoint inhibitors: Recent progress and potential biomarkers. Exp. Mol. Med..

[36] Abdel-Malek Z.A., Swope V.B., Starner R.J., Koikov L., Cassidy P., Leachman S. (2014). Melanocortins and the melanocortin 1 receptor, moving translationally towards melanoma prevention. Arch. Biochem. Biophys..

[37] Shin J.G., Leem S., Kim B., Kim Y., Lee S., Song H.J., Seo J.Y., Park S.G., Won H., Kang N.G. (2021). GWAS Analysis of 17,019 Korean Women Identifies the Variants Associated with Facial Pig-mented Spots. J. Invest. Dermatol..

[38] Zorina-Lichtenwalter K., Lichtenwalter R.N., Zaykin D.V., Parisien M., Gravel S., Bortsov A., Diatchenko L. (2019). A study in scarlet: *MC1R* as the main predictor of red hair and exemplar of the flip-flop effect. Hum. Mol. Genet..

[39] Wu G.S., Luo H.R., Dong C., Mastronardi C., Licinio J., Wong M.-L. (2011). Sequence polymorphisms of *MC1R* gene and their association with depression and antide-pressant response. Psychiatr. Genet..

[40] Sobecki M., Mrouj K., Colinge J., Gerbe F., Jay P., Krasinska L., Dulic V., Fisher D. (2017). Cell-Cycle Regulation Accounts for Variability in *Ki-67* Expression Levels. Cancer Res..

[41] Dikshit A., Jin Y.J., Degan S., Hwang J., Foster M.W., Li C.-Y., Zhang J.Y. (2018). *UBE2N* Promotes Melanoma Growth via *MEK/FRA1/SOX10* Signaling. Cancer Res..

[42] Flori E., Rosati E., Cardinali G., Kovacs D., Bellei B., Picardo M., Maresca V. (2017). The alpha-melanocyte stimulating hormone/peroxisome proliferator activated recep-tor-gamma pathway down-regulates proliferation in melanoma cell lines. J. Exp. Clin. Cancer Res..

[43] Liu J., An H., Yuan W., Feng Q., Chen L., Ma J. (2017). Prognostic Relevance and Function of *MSX2* in Colorectal Cancer. J. Diabetes Res..

[44] Ji M., Li W., He G., Zhu D., Lv S., Tang W., Jian M., Zheng P., Yang L., Qi Z. (2019). *Zinc-α2-glycoprotein 1* promotes EMT in colorectal cancer by filamin A mediated focal adhesion pathway. J. Cancer.

[45] Liu Z.-Y., Tang M.-L., Ning J.-F., Hao Y.-P., Zhou L., Sun X. (2020). Novel octapeptide-DTX prodrugs targeting *MMP-7* as effective agents for the treatment of colorectal cancer with lower systemic toxicity. Eur. J. Med. Chem..

[46] Chen X., Liu Y., Zhang Q., Liu B., Cheng Y., Zhang Y., Sun Y., Liu J. (2021). Exosomal miR-590-3p derived from cancer-associated fibroblasts confers radioresistance in colorectal cancer. Mol. Ther.-Nucleic Acids.

[47] Koslowski M., Türeci Ö., Huber C., Sahin U. (2009). Selective activation of tumor growth-promoting Ca^2+^ channel *MS4A12* in colon cancer by caudal type homeobox transcription factor *CDX2*. Mol. Cancer.

[48] Zhang N., Zhang R., Zou K., Yu W., Wuguo D., Gao Y., Li J., Li M., Tai Y., Huang W. (2017). *Keratin 23* promotes telomerase reverse transcriptase expression and human colorectal cancer growth. Cell Death Dis..

[49] Bury M., Le Calvé B., Lessard F., Maso T.D., Saliba J., Michiels C., Ferbeyre G., Blank V. (2019). *NFE2L3* Controls Colon Cancer Cell Growth through Regulation of *DUX4*, a *CDK1* Inhibitor. Cell Rep..

