# Supplementary material for: MC1R Is a Prognostic Marker and Its Expression Is Correlated with MSI in Colorectal Cancer
Source: Curr Issues Mol Biol. 2021 Oct 11;43(3):1529–47. doi: 10.3390/cimb43030108 (PMC8929037; doi:10.3390/cimb43030108)
Supplement: Supplementary file 1 [file cimb-43-00108-s001.zip › cimb-1361697-supplementary.pdf]

Supplementary materials:

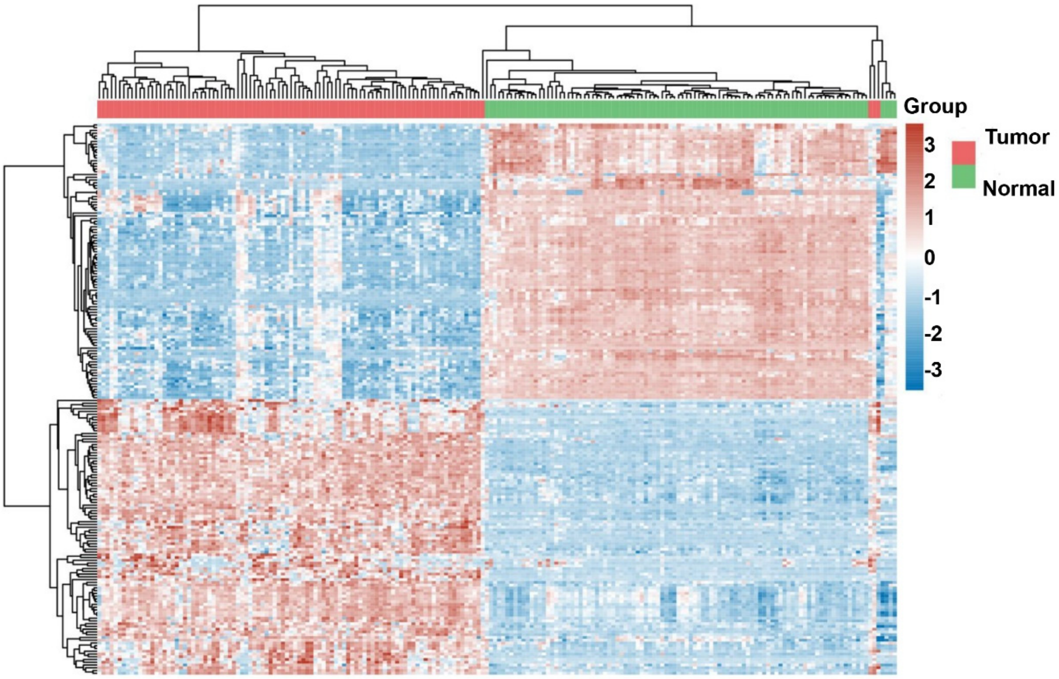

Figure S1: Hierarchical clustering analysis of gene mRNAs, which were differentially expressed between tumor and normal tissues, and the 50 upregulated genes with the largest difference change and the 50 downregulated genes are shown.
